# Antenatal screening and the gendering of genetic responsibility

**DOI:** 10.1186/1742-4755-4-8

**Published:** 2007-09-28

**Authors:** Kate Reed

**Affiliations:** 1 Department of Sociological Studies, University of Sheffield, Elmfield, Northumberland Road, Sheffield S10 2TU, UK

## Abstract

**Background:**

The objective of this study is to explore men's and women's perceptions of antenatal blood screening. The study will assess the impact of these perceptions on decision-making regarding diagnostic testing and selective abortion, and on parental feelings of genetic responsibility. By exploring gender and antenatal screening in this way, the research aims to contribute to our understanding of lay perceptions of genetic screening and increase our knowledge of the decision-making process in screening.

**Research design:**

This qualitative study will be based on semi-structured interviews with twenty pregnant women and twenty male partners in the post-industrial city of Sheffield, UK. All interviews will be taped, transcribed and analysed thematically using NVIVO, a qualitative software package.

**Discussion:**

The findings of this study have relevance to existing debates on the social and ethical implications of reproductive genetics. A better understanding of male and female perceptions of the screening process could improve guidance and practice in antenatal screening and genetic counselling. It will also inform and contribute to the development of theory on gender and genetic screening.

## Background

Within the UK, antenatal blood tests are routinely offered to women in pregnancy during the first NHS dating scan at around 12 weeks gestation. At this time pregnant women are offered a range of tests which include screening for maternal diseases and screening for foetal health. These include tests that identify those who are affected by or at an increased risk of developing a genetic disorder. Such tests include haemoglobinopathy screening for genetic conditions such as sickle cell anaemia and thalassaemia [1]. Blood tests for the hormone human chorionic gonadotrophin (hCG), the protein alpha-feto (AFP) and the protein unconjugated oestriol (uE3) are also offered at this time [2]. This is often known as the 'triple test' and is used to estimate the risk of spina bifida, Down syndrome and anencephaly [3]. Screening takes place at the first dating appointment with the exception of the 'triple test' that takes place after week 14 of the pregnancy. The study on which this protocol is based will be concerned with both haemoglobinopathy screening and with the 'triple test'.

During the screening process the emphasis is on testing women – male partners are tested only where a combined positive male and female test could detect foetal abnormality, such as sickle cell anaemia. Little is known about male partners' views and involvement in antenatal blood screening and their impact on parental feelings of genetic responsibility. Existing research has tended to focus on women as the main recipients of screening and on women's feelings of maternal responsibility [4]. While existing research does acknowledge that factors such as partners, family and peers may play a role in women's screening choices and decisions in antenatal screening, little research focuses in detail on men's roles in screening and antenatal care. For example, little work has been conducted on expectant fatherhood and ultrasoundography [5]. This lack of focus on men's roles can be related to two issues: first, research tends to focus on pregnant women because pregnancy takes place in the female body and thus women are automatically connected to the foetus [6]. Secondly, men are often reticent to take part in research on pregnancy because it is perceived to be a women's issue [7]. However, the nature and optimal level of father involvement continue to interest researchers [8]. Furthermore, researchers often highlight the importance of including men in research on antenatal screening. An American study on amniocentesis found that male partners of pregnant women deeply influenced their partners' decisions to use or refuse antenatal diagnosis and selective abortion [7]. This study highlights the need for more research on gender that includes a focus on both men's and women's perceptions of antenatal screening. A better understanding of the impact of gender on this process will contribute to guidance and practice in antenatal screening and genetic counselling.

Through empirical research with twenty pregnant women and their male partners in Sheffield, UK, this study aims to explore the impact of gender on antenatal blood screening. The study will take an *inductive *approach to research not a *deductive *one, with research problems being explored empirically. The two main research problems to be addressed here are: first, does gender influence choice and decision-making in antenatal screening and, if so, in what ways does it do so? For the purposes of this study gender will be defined as the socially structured differentiation of the sexes [9]. Secondly, does gender affect potential parental feelings of genetic responsibility and, if so, how? What this refers to is an exploration into whether one parent feels more responsible than the other for the genetic status of the foetus. If so, how does this affect decisions made about screening and diagnostic testing?

In order to address these two main research problems, the following subsidiary research questions will be asked within the study:

• How involved are men in the screening process?

• How far do women consult their partners about which tests to opt for and why?

• Do women and men feel equally responsible for the genetic status of the foetus?

• Does the screening process enhance a traditional gendered division of labour – whereby women take the major responsibility for the foetus – or does it challenge traditional gender divisions?

• Finally, how does the gendered nature of the screening process affect any decisions made about the foetus?

In asking such questions, the study aims to explore the impact of gender on antenatal screening, on decision-making and on feelings of parental genetic responsibility.

## Method

In order to assess women's and men's attitudes to antenatal blood screening, a method is needed which generates 'open' data rather then imposing a formalised set of questions [10]. Thus the method chosen for this research is semi-structured interviewing. This method involves posing open ended questions to respondents and following the responses with further questions. This method is often used in qualitative research because it enables the researcher to explore issues in detail [11]. Researchers who take this approach often use a semi-structured guide as a basis for this type of interviews [12]. Within this study an open ended interview schedule will be used. In order to obtain data relating to the research questions stated in this protocol, the interview schedule will be based around screening procedures themselves and the decision-making process. Examples of the types of interview questions to be asked include: which blood tests are opted for and why? Do couples decide which tests to opt for together or does one partner take a more active role in making decisions about screening? Do men and women make decisions about diagnostic tests as a couple or does one partner take more responsibility? The same interview questions will be used for both men and women.

### Sampling

Twenty pregnant women and their male partners will be interviewed within the study, making 40 respondents overall. The primary investigator has substantial experience of conducting small scale studies such as this one [13-15]. Based on previous experience the primary investigator felt that this number of respondents would elicit a substantial but manageable amount of data. However, we will use a flexible approach to sampling, which is common in qualitative research [16]. Should forty respondents yield little data then more interviewing will be conducted. If research categories become saturated at an early stage (see analysis) then fewer interviews will be conducted. This is what is known as an *iterative *approach to research – that is there is a repetitive interplay between the collection and analysis of data [11].

Research on antenatal screening has highlighted the importance of variations according to social class and ethnicity [17,18]. The sample will therefore be stratified according to ethnicity and social class. Ethnicity will be measured here using respondents own self-definition [19]. Social class will be measured using the NS-SEC occupational classification system [20]. In order to access a diverse sample respondents will be recruited from two distinct localities in Sheffield. Ten women and their partners will be recruited from location 1. This is an electoral ward with a non-white population of 30% and where average weekly earnings are below the national average. The second group will be recruited through local midwives in location 2. This is an electoral ward which has a white population of 95% and where the average income is above the national average.

In order to adequately explore the screening process, pregnant women in the study will be at the point of at least 17 weeks' gestation. The reason for selecting this phase of the pregnancy relates to the dates at which blood screening occurs. Haemoglobinopathy screening normally takes place during the first dating scan at 12 weeks with the 'triple test' being offered at around 16 weeks. In order to gain respondents views on these tests women and their partners will be interviewed after they have taken place. To access a wide range of views on screening, respondents will include women and men who are experiencing first, second and third pregnancies. Respondents will have to be between 18 to 40 years of age. These variables will be used as criteria for recruitment and will also be used during data analysis to see if there are any patterns in the effects of gender according to age and number of pregnancy.

### Recruitment

Respondents will be recruited through local NHS community and hospital midwives in Sheffield, UK. Midwives practising in the hospital and in the two relevant community locations will initially be approached by the primary investigator. Information sheets about the research will be given to the midwives and they will be briefed on recruitment criteria. Once their cooperation has been obtained, the midwives will be asked to disseminate this information to pregnant women who meet recruitment criteria and ask women if they and their partners would be willing to participate in the study. If interested in participating, women can either confirm this with their midwife or alternatively contact the research team directly using a stamped self-addressed envelope. The research team will then wait to hear from the midwife and potential respondents and they will then approach those who have left their contact details.

Male partners will be recruited in two different ways. Where possible, they will be recruited during their attendance with partners to screening appointments. However, as men do not always attend routine antenatal appointments, they will also be recruited through pregnant partners. Once pregnant women have agreed to take part in the study, they will be asked if it would be possible to invite their male partners to participate. Another patient information sheet will be given to the women to pass onto the male partner with the same contact options as those outlined above.

As argued by many researchers, it is often difficult to recruit women of colour and working class women in research on pregnant women and antenatal care [17,18]. It has often been argued that qualitative research on women tends to reflect the views of white middle class women because they tend to volunteer to participate in research more than any other group. This often leads to a self-selecting and biased sample. In order to try and address this issue social researchers tend to advocate the use of more labour intensive strategies such as verbal face-to-face contact, ethnicity matching and snowballing [21]. Within this study on gender and antenatal screening, two different geographical areas will be chosen in order to avoid a self-selecting sample. Should it prove difficult to elicit a diverse sample in this study, then more labour intensive strategies will be employed. This would probably involve contacting various pregnancy groups and local government initiatives in the relevant locations.

### Data collection

Data will be collected by the primary investigator and one researcher through the use of an agreed interview schedule. The interviews will be conducted in antenatal clinics. Where possible interviews will take place in private rooms within clinics. If respondents are not happy with being interviewed in this environment, interviews will take place in respondents' homes or in a location suitable to them.

All interviews will last approximately 1 hour and will be tape-recorded. As with much social science research, issues may be raised relating to the reliability and validity of the project [11]. Semi-structured interviews, because of their subjective nature can be affected by interviewer bias. Issues of reliability are also called into question due to the fact that two people will be interviewing on this project rather than one person which may lend it further to interviewer bias. Furthermore, the subjective nature of the research also brings into question issues of validity as research accounts may be subject to misinterpretation and misrepresentation by the researcher. Within the study these problems will be counteracted by firstly providing a very clear interview schedule for both researchers to follow. The same questions will be asked of all respondents. In order to ensure validity, the interviewees' answers will be relayed back to them for verification after each interview [22]. In order to maintain reliability using two interviewers, some joint interviews will be conducted with both researchers present (at the start of fieldwork).

Ethical approval to conduct the study was granted by the **South Sheffield Research Ethics Committee **on 08/10/2006 Reference number 06/Q2305/155. All respondents will provide written consent prior to each interview. Respondents and interview transcripts will all remain anonymous.

### Transcription and analysis of data

Having collected the data, it will be transcribed and analysed. In order to explore potential differences in social class and ethnicity the data from the two different locations will be analysed separately and then compared. The data will be analysed drawing on a grounded theory approach which means that the research will not start with a hypothesis, but rather, data will be used to inform existing social theories and also to develop new ones [23]. The process of data analysis will take several stages: First, interview transcripts will be reviewed and coded. Codes serve as shorthand devices to label, separate, compile and organize data [24]. NVIVO, a qualitative software package will be used to assist the coding process through the identification of commonly used words and phrases in the transcripts. After coding, data will then be organized into themes and categories. Social theories will then be developed using these themes and research categories.

### Time plan for the study

Data collection will take place between January 2007 and 31^st ^July 2007. Data analysis will take place between August 2007 and October 31^st ^2007 with the dissemination of findings occurring shortly afterward.

## Discussion

It is important to remember that this is only a small scale study and therefore the findings are not generalisable to the population at large. However, the findings of the research will offer a modest contribution to academic debates in the area of reproductive genetics. There has been much debate about the social and ethical issues of genetic antenatal screening, particularly because of the spectre of eugenics that hangs over genetic testing [25]. This research project will contribute to our understanding of the social implications of genetic screening by evaluating lay perceptions of men and women's roles in antenatal screening. Furthermore, the concepts and categories developed from the research will be used to inform existing social theories on gender, genetics and screening.

The research also has implications for health practitioners. Genetic counselling in antenatal screening is generally considered to be of a high standard in the UK. According to recent reports however, the screening setting is often highly pressurized with too much emphasis on routine and not enough time to explore patient feelings towards screening and diagnostic tests [26]. Through increasing our understanding of people's perceptions of genetic testing, this qualitative study aims to offer a modest contribution towards enhancing screening practice. In particular, the study will provide us with a better understanding of screening among diverse populations. Thus it will enhance sensitivity to diverse populations in screening practices and genetic counselling.

The research is also relevant to policy-makers who are attempting to make screening procedures more sensitive to the needs of potential parents.

## Competing interests

The author(s) declare that they have no competing interests.

## Authors' contributions

KR is the sole author of this article.
